# Shining Damaged Hearts: Immunotherapy-Related Cardiotoxicity in the Spotlight of Nuclear Cardiology

**DOI:** 10.3390/ijms23073802

**Published:** 2022-03-30

**Authors:** David Kersting, Stephan Settelmeier, Ilektra-Antonia Mavroeidi, Ken Herrmann, Robert Seifert, Christoph Rischpler

**Affiliations:** 1 Department of Nuclear Medicine, University Hospital Essen, West German Cancer Center (WTZ), University of Duisburg-Essen, 45147 Essen, Germany; ken.herrmann@uk-essen.de (K.H.); robert.seifert@uk-essen.de (R.S.); christoph.rischpler@uk-essen.de (C.R.); 2 German Cancer Consortium (DKTK, Partner Site Essen/Düsseldorf), 45147 Essen, Germany; ilektraantonia.mavroeidi@uk-essen.de; 3 Department of Cardiology and Vascular Medicine, University Hospital Essen, West German Heart and Vascular Center, University of Duisburg-Essen, 45147 Essen, Germany; stephan.settelmeier@uk-essen.de; 4 Clinic for Internal Medicine (Tumor Research), University Hospital Essen, West German Cancer Center (WTZ), University of Duisburg-Essen, 45147 Essen, Germany

**Keywords:** molecular imaging, PET, SPECT, cardiotoxicity, immunotherapy, nuclear medicine, nuclear cardiology, cardio-oncology

## Abstract

The emerging use of immunotherapies in cancer treatment increases the risk of immunotherapy-related cardiotoxicity. In contrast to conventional chemotherapy, these novel therapies have expanded the forms and presentations of cardiovascular damage to a broad spectrum from asymptomatic changes to fulminant short- and long-term complications in terms of cardiomyopathy, arrythmia, and vascular disease. In cancer patients and, particularly, cancer patients undergoing (immune-)therapy, cardio-oncological monitoring is a complex interplay between pretherapeutic risk assessment, identification of impending cardiotoxicity, and post-therapeutic surveillance. For these purposes, the cardio-oncologist can revert to a broad spectrum of nuclear cardiological diagnostic workup. The most promising commonly used nuclear medicine imaging techniques in relation to immunotherapy will be discussed in this review article with a special focus on the continuous development of highly specific molecular markers and steadily improving methods of image generation. The review closes with an outlook on possible new developments of molecular imaging and advanced image evaluation techniques in this exciting and increasingly growing field of immunotherapy-related cardiotoxicity.

## 1. Introduction

The emerging use of novel immunotherapies in cancer treatment increases the risk of immunotherapy-related cardiovascular side effects. Cardiotoxic damage ranges from asymptomatic changes to fulminant short- and long-term complications, including cardiomyopathies, arrythmias, and vascular disease [1,2,3,4,5]. In contrast to conventional chemotherapy, the use of targeted immunotherapies has expanded the forms and presentations of cardiovascular toxicity [4]. Accompanied by increasing long-term survival rates of cancer patients, integrated management and care of cardiovascular disease through cancer and cancer therapy by cardio-oncologists become important to reduce cardiovascular side effects which affect prognosis and quality of life [6].

Immunotherapies for cancer treatment follow passive or active strategies. While in passive immunotherapy, monoclonal antibodies (mAbs) targeting growth receptors, immune checkpoint inhibition, or bispecific T cell engagement are used, active strategies contain especially chimeric antigen receptor (CAR)-T cell transfer [7,8]. All of them can be associated with immunotherapy-related cardiovascular side effects. Monoclonal antibodies targeting ERB epitopes are widely used in the treatment of breast cancer (Anti-HER2/neu) and squamous cell carcinoma/colorectal carcinoma (EGFR) and can cause myocardial dysfunction, heart failure, hypertension, and arrythmia [7,9,10]. The risk of cardiotoxicity even increases as conventional chemotherapeutics are used concomitantly in those treatment regimens [11]. Inhibition of vascular endothelial growth factor (VEGF) signaling using anti-VEGF mAbs can induce arterial hypertension and lead to thromboembolic complications [12]. Inhibition of protein kinases such as MEK and BRAF have been associated with left ventricular dysfunction and a higher risk of pulmonary embolism due to thromboembolic complications [13]. Immune checkpoint inhibitors targeting programed death-1 (PD-1) or cytotoxic T-lymphocyte antigen-4 (CTLA-4) can induce severe myocarditis and further increase the risk of major cardiovascular events including acute coronary syndromes and arrythmias [14,15,16,17]. Bispecific T cell engagement as used in B-cell acute lymphoblastic leukemia can cause severe cardiovascular side effects presenting with fatal heart failure due to massive cytokine release syndrome [7,12,18,19]. In addition, CAR-T cell therapy as a revolutionary advance in the therapy of hematologic malignancies can severely affect the cardiovascular system by a severe inflammatory response condition due to massive cytokine release syndrome [8,20,21].

Cardio-oncological monitoring in cancer patients and in patients undergoing cancer therapy is a complex interplay between pretherapeutic assessment and risk identification, therapeutic monitoring with identification and evaluation of impending cardiotoxicity, and its therapeutic management and post-therapeutic surveillance to identify late-onset cardiotoxicity [2]. Recent position statements therefore provided detailed recommendations for individual patients depending on risk assessment and therapy-related risk factors [1,2,3,4].

In addition to physical examination and baseline cardiological assessment using echocardiography, the cardio-oncologist can revert to a broad spectrum of nuclear cardiological diagnostic workup [22,23]. Many nuclear medicine imaging techniques can be used to detect cardiovascular toxicity of which the most promising and commonly used modalities will be discussed in this review article. Due to the novelty of the topic, their application in the field of immunotherapy-related cardiotoxicity has so far been limited to few described individual applications. However, with more insight into pathophysiology, targeted diagnostic or even theranostic approaches may gain importance in the future. The article, therefore, first presents the nuclear cardiological examination techniques and then, for the methods that are considered most promising, transfers their application potential to immunotherapy-induced effects. The review closes with an outlook to possible new developments of molecular imaging and advanced image evaluation techniques in this exciting and increasingly growing field of immunotherapy-related cardiotoxicity.

## 2. Molecular Imaging: Visualization of Metabolic Pathways and Body Function

The typical molecular imaging techniques in nuclear medicine are used to visualize metabolic pathways or other aspects of human body functions via imaging of the in vivo distribution of radiolabeled substances. Radionuclides are bound to molecules which, after their (predominantly intravenous) application can interact with various molecules inside the human body or be used for imaging of their distribution without direct binding to target molecules (e.g., imaging of myocardial perfusion). In principle, the radionuclide is crucial for generation of images, while the molecule bound to it guides the compound to its target position within the body. Following the tracer principle [24,25], only very small amounts of compounds are applied, which, on the one hand, are sufficient to visualize the investigated body function, but, on the other hand, have no biological effect.

Two-dimensional planar scintigraphy, three-dimensional single photon emission computed tomography (SPECT) and three-dimensional positron emission tomography (PET) techniques are currently used for nuclear medicine imaging in clinical routine practice. For each of these imaging modalities, many different tracers for various target structures are available, some of which are firmly established in routine clinical practice and others of which are being evaluated experimentally or used in the context of specific scientific questions.

### 2.1. Molecular Imaging Tracers and Target Structures

Typical oncological applications, which commonly exploit the possibility of whole-body imaging, include primary staging and re-staging to identify metastases (in patients with known primary) or search for primary tumors (in patients with cancer of unknown primary origin) [26,27,28,29,30,31]. Here, the molecular targets are, for example, glucose metabolism, bone remodeling [32,33,34,35], or tumor-specific receptors and molecules involved in metabolic pathways of carcinogenesis such as somatostatin receptors [36,37,38,39,40,41,42] (for imaging neuroendocrine tumors) or prostate-specific membrane antigen [43,44,45,46,47,48,49,50] (for imaging prostate carcinoma). These specific oncological targets can additionally enable the possibility of radionuclide therapies using similar target structures but beta-minus-emitting radionuclides such as ^131^I, ^177^Lu, or ^90^Y. These deliver their energy to the surrounding tissue in short distance to evoke therapeutical effects in the context of individual theranostic concepts [51,52,53,54,55,56,57,58,59,60,61,62].

In addition, in non-oncological patients, molecular imaging investigations of individual organ systems are firmly established. These include evaluation of thyroid function and assessment of thyroid nodules [63,64,65,66,67], examinations of the brain to visualize amyloid deposits for diagnosis of Alzheimer’s disease [68,69,70,71] or presynaptic dopamine transporters for diagnosis of Parkinson’s disease [72,73,74,75], or evaluation of lung ventilation and perfusion for diagnosis of pulmonary artery embolism [76,77,78,79].

Finally, the examination of the heart is an integral part of clinical routine in nuclear medicine. In the context of the increasing use of immunotherapies for cancer treatment and the occurrence of cardiotoxic side effects, molecular imaging modalities are also increasingly used to evaluate cardiac function in this patient population. The most promising methods, according to the authors’ opinion, are discussed in this review. First, a brief overview of technological aspects is given for the most important nuclear cardiologist methods of image generation.

### 2.2. Planar Scintigraphy and Single Photon Emission Computed Tomography

Planar scintigraphy and SPECT are imaging modalities that are based on the usage of gamma emitting radionuclides [80,81]. To generate images, emitted photons are detected using gamma cameras (planar imaging) or tomography systems (SPECT). The processing of additionally acquired computed tomography images (SPECT/CT) enables an improvement of image quality by attenuation correction as well as facilitating the anatomical assignment of the molecular imaging signal [82].

The gamma emitting radionuclide that is predominantly used in routine practice is ^99m^Tc, as it exhibits particularly favorable properties for image generation and practicability in daily use [83]. These include, inter alia, a short half-life of 6 h as well as the possibility of obtaining the radionuclide from an inhouse generator and binding it to target molecules without the need for a radiochemistry department inside the clinic or outbound practice. In addition, for some tracers, the radionuclide ^123^I is used, which has a longer half-life of about 13 h [84].

With regards to drug-related cardiotoxicity, several radiotracers for planar scintigraphy/SPECT imaging are available which can be used to visualize different parameters characterizing cardiac function and damage. These include (time-resolved) visualization of ventricle morphology, examination of myocardial perfusion, and indirect visualization of myocardial damage via imaging of the sympathetic innervation system of the heart.

### 2.3. Positron Emission Tomography

In contrast to planar scintigraphy and SPECT, PET imaging uses positron emitters as radionuclides. The collision of positrons and electrons in the target tissue produces two annihilation photons which are emitted at an angle of about 180° to each other. The PET imaging technique uses only coincidence events (if two photons are detected at opposite positions of the detector rings) to generate images. Therefore, the number of random detections (arising, for example, from background radiation) is reduced and a higher image quality and lower image noise compared to SPECT imaging are achieved.

In current practice, PET is performed exclusively in hybrid imaging technique, i.e., in combination with magnetic resonance tomography (PET/MR) or computed tomography (PET/CT). Like for SPECT/CT imaging, the reasons are on the one hand technical: the additional morphological imaging modality can be used for attenuation correction, which increases image quality and enables precise quantification of tracer uptake. On the other hand, it allows for anatomical assignment of structures and the simultaneous possibility of radiological co-assessment of pathologies with or without increased tracer accumulation.

The two radionuclides that are most frequently used in clinical routine are ^68^Ga and ^18^F [25]. These are widely available and have some features useful for routine clinical use such as a short half-life and a high proportion of positrons in their decay process leading to a high PET image quality. For imaging of cardiotoxicity, mostly glucose metabolism PET imaging after application of ^18^F-FDG has been used and also been applied to immunotherapy patients. Recently, examinations using ^68^Ga-DOTATOC/DOTATATE, which bind to somatostatin receptors, or ^68^Ga-labeled fibroblast activating protein inhibitors (^68^Ga-FAPIs), which target the fibroblast activating protein (FAP), were also described. Therefore, these tracers were selected for a more detailed discussion. For all three tracers, the approaches aim at using the improved image quality of PET for an early detection of cardiotoxicity. Moreover, imaging in PET technique opens the possibility of PET/MR hybrid imaging. This can allow for combined assessment of subtle morphological and functional abnormalities that might lead to very early detection of cardiotoxicity [85].

In this review, for the most promising imaging modalities, possible benefits and application scenarios will be discussed. Due to the limited applications published to date in the field of immunotherapy-related cardiotoxicity, aspects that have been investigated when used for related questions will be transferred to this topic. The examination modalities are organized by molecular target structures and presented in descending order starting with the most promising (in the authors’ opinion) modality.

## 3. Imaging of Glucose Consumption

For the imaging of glucose metabolism, ^18^F-FDG PET is performed. With proper preparation of the patient, an increased glucose metabolism can indicate myocarditis or ischemic myocardium as well as vascular toxicity [86,87,88]. These pathologies are linked to immunotherapy, making ^18^F-FDG PET imaging a suitable tool for early detection of adverse effects during therapy which stands out due to its high image quality and the possibility of detection of subtle abnormalities. Additionally, ^18^F-FDG PET is often habitually performed in oncological patients for re-staging and assessment of therapy success; therefore, the possibility of a simultaneous examination of the heart with a single protocol can arise or cardiac readouts may be performed from routine oncological scans. This might allow for a specific screening for cardiotoxic effects and leads the authors to consider ^18^F-FDG imaging as the most promising imaging modality in this review.

In the pathophysiological development of chemotherapy-related cardiotoxicity, cardiac glucose metabolism can be increased leading to a possible early detection by ^18^F-FDG PET [89]. In the context of immunotherapy-related cardiotoxicity, several reports describe an application for investigation of immune checkpoint inhibitor-related myocarditis [11,90,91,92,93]. These hint at a possibility of early detection and possible improvements compared to cardiac MR [94]. Moreover, ^18^F-FDG is an established and commercialized PET tracer that is available at many centers.

**Figure 1:**
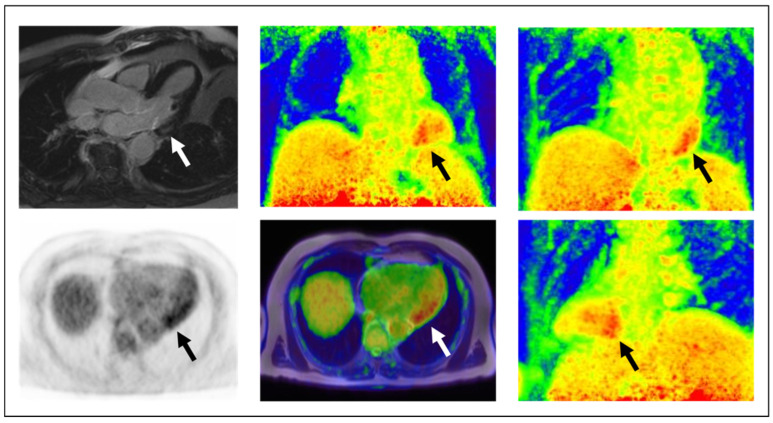
Example of a metastatic malignant melanoma patient under nivolumab treatment in whom checkpoint inhibitor-related myocarditis was verified by ^18^F-FDG PET/MR in our institution. Clockwise from upper-left: Late-phase MR, maximum-intensity-projection PET images from three different angles (anterior, posterior, left-posterior view), axial fusion PET/MR, axial PET. Hybrid imaging showed a hypertrophied normal-sized left ventricle with normal left ventricular ejection fraction and with circumscribed left-ventricular midmyocardial late gadolinium enhancement (infero-latero-basally, white arrow in upper-left image), mild edema, and increased ^18^F-FDG accumulation (black/white arrows in PET image) which was indicative of active myocarditis.

Technically, ^18^F-FDG accumulates in cardiomyocytes after uptake through GLUT receptors and intracellular retention following phosphorylation by hexokinase [25]. For suppression of the PET signal from physiological myocardial glucose consumption, adequate patient preparation must be performed that aims at shifting myocardial energy consumption to free fatty acids. Typical protocols include prolonged fasting (for at least 12 h), carbohydrate restriction (Atkins diet) for at least one day prior to scanning, fatty meals, and loading with unfractionated heparin (at about 15 min before ^18^F-FDG injection) [95]. Figure 1 shows an example of a patient with metastatic malignant melanoma under adjuvant anti-PD1 immune checkpoint inhibition treatment with nivolumab. The patient underwent ^18^F-FDG PET/MR as immune checkpoint inhibitor-related myocarditis was suspected due to elevated troponin. Eventually, active myocarditis was verified by PET/MR imaging showing the possibility to perform the examination in immunotherapy-related cardiotoxicity patients.

Despite the advantages of ^18^F-FDG PET for imaging of immunotherapy-related toxicity, some opinions point to a limited diagnostic value of the technique [96]. In addition, cardiac ^18^F-FDG PET uptake can be non-specific and disregarding the laborious patient preparation protocol may prevent diagnostic validity [93]. Therefore, further systematic investigation is needed to identify patient groups in whom ^18^F-FDG PET should be performed as imaging modality of choice and to review PET investigations using other tracers that may have further specific advantages for imaging of immunotherapy-related toxicity.

## 4. Imaging of Cardiac Remodeling and SSTR-Expression

One of these novel PET imaging modalities that can broaden the applicability of PET imaging for immunotherapy-related cardiotoxicity is the recently introduced ^68^Ga-FAPI PET. Several reports describe the applicability of the technique, which is also used for oncologic imaging [97,98] and subsequent radionuclide therapy in a theranostic approach [99], for imaging of cardiac fibroblast activation as an early sign of cardiac damage that might be induced by immunotherapy-related cardiotoxicity [100,101,102]. Thus, early detection might be possible [101] using a highly specific target that does not exhibit the shortcomings of ^18^F-FDG PET. Moreover, also for ^68^Ga-FAPI PET the possibility of simultaneous oncological staging and cardiac assessment in a single examination emphasizes its significance as a highly promising tool. However, ^68^Ga-FAPI has not yet been approved and examinations are performed as part of clinical trials or as compassionate use. Therefore, its availability is still limited to large centers and further developments have to be awaited before widespread use can be recommended.

**Figure 2:**
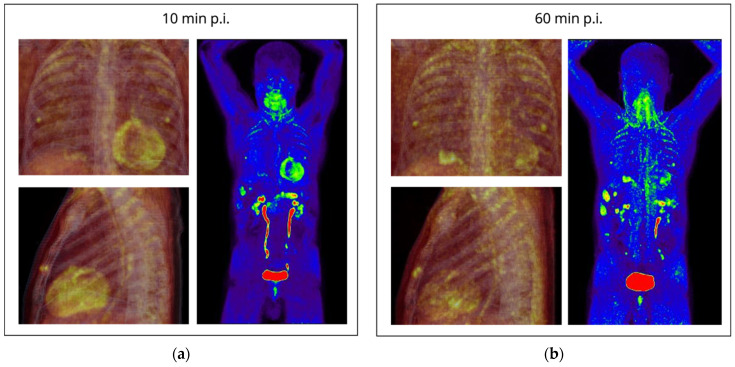
Example of a metastatic pancreatic ductal adenocarcinoma patient under distinct chemotherapy regimens (gemcitabine/nab-paclitaxel followed by modified FOLFIRINOX). (**a**) Early ^68^Ga-FAPI PET images 10 min p.i. (**b**) Late ^68^Ga-FAPI PET images 60 min p.i. Clockwise from upper-left: PET/CT fusion anterior view, PET/CT fusion lateral view, PET maximum-intensity-projection anterior view. Hybrid imaging shows the primary pancreatic carcinoma, multiple liver metastases, and peritoneal nodules (indicative of peritoneal carcinomatosis), each with intense FAP-expression. Chemotherapy-related cardiotoxicity was suspected due to increased cardiac FAP-expression (emphasized in early images p.i.) and incomplete cardiac wash-out (observed in late images). PET/CT images were acquired at our institution.

Biologically, ^68^Ga-FAPI targets FAP, a cell-surface serine protease that acts on various hormones and extracellular matrix components [103]. FAP is expressed rarely in healthy adult tissues. However, it is highly upregulated in a wide variety of cancers at sites of active tissue remodeling and is often used as a marker for pro-tumorigenic stroma (cancer mesenchymal stem cells, cancer-associated fibroblasts, sarcoma, and melanoma cells), wound healing, and fibrosis [104]. Moreover, FAP shows an overexpression in benign remodeling processes as they can occur in the context of immunotherapy-related cardiac damage [93,100,105] and after myocardial infarction [106]. After myocardial infarction, fibroblasts undergo dynamic phenotypic changes and differentiate into collagen-secreting proto-myofibroblasts, which can further differentiate into mature myofibroblasts. Activated fibroblasts migrate into the injured myocardium and contribute to tissue replacement, thereby helping to preserve the structural integrity of the infarcted heart, which elicits reactive and interstitial fibrosis and hence decreases cardiac contractility [106]. This pathophysiological process might in part be comparable to remodeling processes after immunotherapy-related damage to cardiomyocytes. Moreover, FAP is upregulated in cardiomyopathies [107]. Figure 2 shows an example of a pancreatic ductal adenocarcinoma patient under distinct chemotherapy regimens who displayed increased cardiac ^68^Ga-FAPI uptake as a possible sign of chemotherapy-related cardiotoxity.

Moreover, somatostatin receptor (SSTR) PET that was previously identified as promising tool for imaging of myocarditis [108] was recently applied for early detection of immune checkpoint inhibitor-related myocarditis. A case series [109] and a case report [102] describe applicability for both ^68^Ga-DOTATOC and ^68^Ga-DOTATATE (both tracers target SSTR but show differences in their affinity to different SSTR subtypes). Earlier detection of cardiac damage than by cardiac MR is a possible benefit and, compared to ^18^F-FDG PET, no laborious patient preparation is necessary [109]. Future studies are necessary to confirm these results which hint at an identification of somatostatin receptor PET as additional modality for imaging of immunotherapy-related cardiotoxicity. However, its applicability for oncological staging is limited to somatostatin receptor expressing malignancies which precludes broad use for simultaneous staging and cardiac assessment. On the other hand, ^68^Ga-DOTATOC/DOTATATE are commercialized and, therefore, available at many centers.

The small number of published reports on the applicability of non-FDG PET imaging for immunotherapy-related cardiotoxicity underlines that further systematic investigations are necessary before a widespread clinical application can be achieved. Moreover, future studies might also introduce different tracers targeting other specific molecules in the interaction of immunotherapy and myocardium and advanced methods of image evaluation.

## 5. Imaging of Myocardial Damage

A different diagnostic tool for early detection of myocardial damage that can be applied in scintigraphy, SPECT, and PET techniques is imaging of cardiac sympathetic innervation. For the commonly performed imaging in scintigraphy or SPECT technique, ^123^I-meta-iodobenzylguanidine (^123^I-MIBG) is applied, which is a norepinephrine analog and therefore a tracer for sympathetic neuron integrity and function. The dysfunction of the autonomous nervous system contributes to the pathophysiology of heart failure and ^123^I-MIBG imaging has already found its way into clinical examination of heart failure patients [110,111].

As chemotherapy-induced damage to the myocardial beta-adrenergic system might contribute to the pathophysiology of cardiotoxicity [112,113] and preclinical studies hint at an earlier detection of chemotherapy-related cardiotoxicity by a decrease in ^123^I-MIBG uptake than, for example, by a decline in left ventricular ejection fraction (LVEF) [114], ^123^I-MIBG SPECT imaging is a promising technique for early detection of chemotherapy-related cardiotoxicity. Moreover, ^123^I-MIBG is a commercialized tracer available at many centers. However, it has not yet been established for clinical routine imaging in this patient cohort.

**Figure 3:**
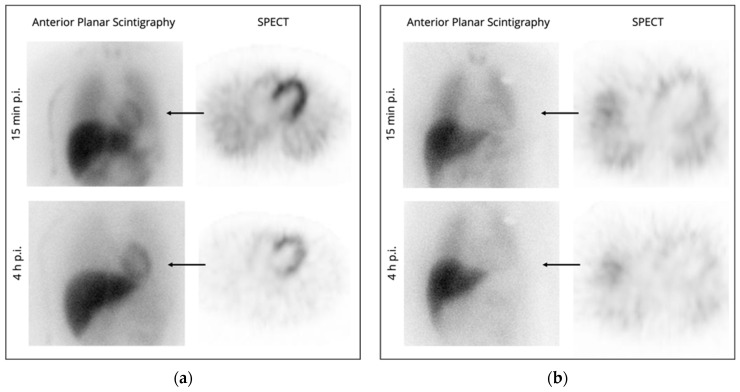
(**a**) Example for a physiological result in ^123^I-MIBG imaging: Physiological sympathetic cardiac innervation (HMR late: 2.1, normal range: 2.0–2.4; physiological washout of 14%). (**b**) Example for a pathologic result: Decreased sympathetic cardiac innervation (HMR late: 1.3; increased washout of 28%). HMR: heart-to-mediastinum ratio. Images were acquired at our institution.

Most of the literature on the use of ^123^I-MIBG imaging for evaluating patients with heart failure is based on measurements from anterior planar scintigraphy images with cardiac uptake quantified in terms of the heart-to-mediastinum ratio (HMR) and the washout rate between early and late images [115]. ^123^I-MIBG SPECT has been shown to yield complementary results to planar imaging in patients with heart failure after acute myocardial infarction [115]. Figure 3 depicts examples of physiological and pathologic ^123^I-MIBG planar scintigraphy and SPECT imaging.

To date, the application of the technique has not explicitly been described for imaging of immunotherapy-related cardiotoxic effects. Given that for immunotherapy, like for chemotherapy, cardiotoxic effects to the sympathetic innervation seem possible, ^123^I-MIBG imaging might also be a promising technique for early detection of cardiac damage in this patient group. Future systematic investigations are necessary to elucidate possible benefits. These might also focus on the application of imaging of cardiac innervation in PET technique using ^124^I-MIBG, 18F-dihydroxyphenylalanine (^18^F-DOPA) [116], or ^18^F-flubrobenguane which [117] which shows a similar behavior to MIBG. In this way, a higher image quality can be achieved, which could also improve the possibility of early detection of immunotherapy-related cardiotoxic effects. However, these tracers are currently only used in clinical trials or small patient cohorts and are not widely available.

## 6. Investigation of Myocardial Perfusion and Viability

Myocardial perfusion imaging is one of the most frequently performed molecular imaging procedures for evaluation of the heart. The examination is typically performed both in rest and after physical stress to investigate perfusion of the myocardium in conditions of reversible ischemia [118]. The comparison of stress and rest images allows detection of stress-induced perfusion defects as an indicator for coronary artery disease [119]. Moreover, myocardial viability can be evaluated [120] and the LVEF can be estimated in electrocardiogram-gated examinations [118]. Imaging can either be performed using cardiac-centered gamma cameras [118] or in SPECT(/CT) technique [121].

**Figure 4:**
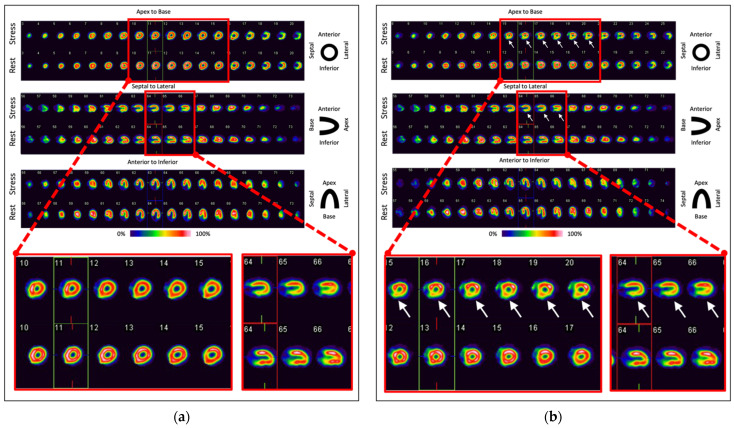
(**a**) Example for a physiological result in myocardial perfusion SPECT: No stress-induced perfusion defects, no ischemia, normal LVEF in stress and rest conditions (≥55%). (**b**) Example for a pathologic result: Stress-induced perfusion defects of the posterior wall (white arrows, ischemia: 10% of left-ventricular myocardium), normal LVEF in stress and rest conditions (≥55%). Images were acquired at our institution.

Typically, the ^99m^Tc-labeled tracers ^99m^Tc-sestamibi and ^99m^Tc-Tetrofosmin are used for myocardial perfusion imaging. These accumulate in cardiac mitochondria and are not washed out from myocardial tissue after their uptake [122,123,124,125]. Alternatively, the thallium isotope ^201^Tl can be used, which mimics potassium in physiological processes (transport into myocardiocytes by Na-K-ATPase and subsequent wash-out), the uptake process correlates linearly with myocardial perfusion [122]. However, ^201^Tl myocardial perfusion imaging has a higher radiation exposure to patients than ^99m^Tc-sestamibi and ^99m^Tc-Tetrofosmin [126] and can, therefore, currently be considered a second-line option. For an examination under stress conditions, either ergometric stress can be performed or pharmacological stress can be evoked by application of vasodilators such as the cardiac adenosine receptor agonists dipyridamole, adenosine, or regadenoson or by administration of the catecholamine dobutamine prior to injection of the radiopharmaceutical. Regadenoson can be recommended in most situations because of the drug’s safety and adverse event profile and the simplified study protocol compared with the other agents [127]. Figure 4 shows examples of physiological and pathological myocardial perfusion SPECT results.

In oncological patients, myocardial perfusion imaging can be performed for pretherapeutic risk evaluation and to assess or monitor treatment-related cardiotoxic effects during or after immunotherapy. Myocardial perfusion imaging enables evaluation of LVEF and perfusion defects in one single examination. Patients might benefit from this simultaneous assessment, as acute coronary syndrome and (worsening of) atherosclerotic vascular disease are described complications of immune checkpoint inhibitor treatment [128,129]. Furthermore, in patients receiving anti-PD1 therapy left ventricular function was impaired in response to stress [130] making stress testing a promising examination technique.

The evaluation of myocardial perfusion imaging is highly standardized, approved, and the applied tracers are commercialized and widely available. Compared to echocardiography, assessment of LVEF by myocardial perfusion imaging is less susceptible to external factors [131]; compared to cardiac MR, the technique is advantageous in cost-effectiveness and patients who can or must not undergo MR imaging [131]. Therefore, myocardial perfusion imaging can potentially lead to early diagnosis and timely beginning of cardiac therapy and/or adjustment of immunotherapy, and several reports describe application of myocardial perfusion imaging in patients with immunotherapy-related cardiotoxicity [132,133,134]. As proposed, the technique was used for monitoring of cardiac function during immunotherapy treatment and for clarification of echocardiography results. However, due to cardiac reserve, myocardial damage may occur well before a measurable decrease in LVEF. Therefore, monitoring this parameter might not be the optimal technique for early detection of patients with therapy-related cardiotoxicity [112,135] and the use of more promising examination techniques such as the previously described PET imaging modalities may be useful.

Of note, myocardial perfusion imaging can also be performed in PET technique using, for example, H_2_^15^O, ^13^NH_3_, or ^82^Rb, with the benefit of quantitative image evaluation [127]. However, these examinations are technically demanding, and the required radiopharmaceuticals are not available in many centers. Systematic applications of myocardial perfusion PET in the context of oncologic treatment have only been described in rare reports and preclinical studies with most of them in the context of radiotherapy and none of them covering immunotherapy patients [90,136,137,138,139,140]. In this manuscript, we therefore refrain from a detailed description of the technique which can, alternatively, be found in various previously published review articles, for example, by Driessen et al. [141] or by Nakazato et al. [142].

## 7. Assessment of Ventricular Function and Chamber Morphology

Equilibrium radionuclide angiography (ERNA) can be used to visualize chamber morphology, determine ventricular volumes, and evaluate ventricular wall motion. From electrocardiogram-gated ERNA data, systolic and diastolic volumes can be estimated and the LVEF can be calculated [143,144]. Monitoring of LVEF can be considered a clinical standard to assess the functional impact of immunotherapy-related cardiotoxicity, as heart failure was described as an adverse event of different immunotherapeutic treatment regimens including nivolumab [145], nivolumab plus ipilimumab [146], or pembrolizumab [147]. ERNA has not yet been described in immunotherapy-related cardiotoxicity patients but, for example, in breast cancer patients that are treated with the monoclonal antibody trastuzumab [148], which targets HER2/neu receptor [149]. Cardiac dysfunction, in particular congestive heart failure, is a typical adverse event in treatment with trastuzumab [131,150] and requires monitoring of LVEF.

**Figure 5:**
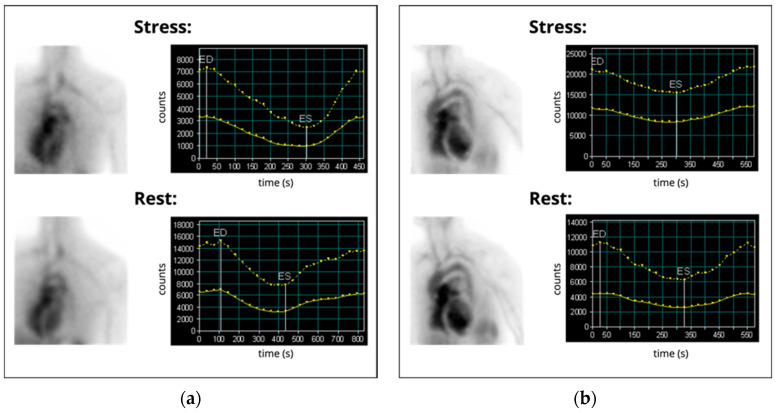
(**a**) Example of a physiological result in planar scintigraphy equilibrium radionuclide angiography: Normal LVEF and regular LVEF reaction to stress (rest: 56%, stress: 73%). (**b**) Example for a pathologic result: Decreased LFEF and inadequate LVEF reaction to stress (rest: 26%, stress: 24%). ED: end-diastolic, ES: end-systolic. Images were acquired at our institution.

Technically, ERNA is a blood pool scintigraphy, for which commonly patient erythrocytes are labeled with ^99m^Tc. Labeling can be performed in vivo, in vitro, or in a mixed in vivo/in vitro method [143]. As an alternative to labeling of erythrocytes, ^99m^Tc-labeled human serum albumin can be used but exhibits higher background activity [151]. The examination is typically performed in planar imaging technique. Alternatively, SPECT imaging is possible and allows for additional investigation of right ventricular function [152]. ERNA is performed in rest and after ergometric exercise to examine an adequate increase in LVEF in response to physical stress [153]. Figure 5 shows examples of physiological and pathologic ERNA results.

ERNA shows high precision and repeatability [154,155] and fewer variations depending on external circumstances than echocardiography (e.g., observer-dependent factors or quality of echogenic window) [131]. Moreover, the imaging technique is well established and widely available. Therefore, in the context of immunotherapy-related cardiotoxicity, ERNA may be particularly beneficial for pretreatment assessment and to monitor cardiac function during therapy (including post-therapeutic surveillance). An application scenario can be patients with borderline LVEF in echocardiography, in whom accurate diagnosis influences further therapy management [1,156]. Moreover, the high repeatability might facilitate systemic evaluations in multi-centric clinical trials. Compared to cardiac MR, which is also a highly accurate and sophisticated imaging technique to evaluate even small cardiac damage, advantages of ERNA include a higher cost-effectiveness [157] and the applicability to patients who must not undergo MR after implementation of cardiac devices. A drawback might be a limited applicability for early detection of cardiac damage which, from the authors’ point of view, makes the previously presented (PET) imaging modalities more promising options in the field of immunotherapy-related cardiotoxicity.

## 8. Future Trends

Future developments in molecular imaging related to the cardiotoxicity of immunomodulatory drugs may include the investigation of new molecular targets. These will particularly benefit from the higher image quality of PET imaging compared to conventional planar scintigraphy or SPECT techniques. In addition, imaging technology itself is subject to continuous technical development, both on the software and on the hardware side. New developments may significantly improve the image quality and, therefore, also the possibilities of application, for example, regarding detection of low activity concentrations as they may appear in imaging of new molecular targets. In addition, higher image quality also facilitates the application of advanced image analysis techniques. These include, for example, the analysis of time-resolved dynamic data. In addition, the study of molecular imaging data using artificial intelligence techniques is spreading. These methods, on the one hand, enable the analysis of large amounts of data and, on the other hand, can allow the determination of parameters within medical images that are not accessible to the human eye.

### 8.1. New Molecular Targets

The use of novel immunotherapeutic drugs may be accompanied by the possibility of specific labeling of these compounds for PET imaging and visualization of their distribution within the human body. For some of these substances, such imaging possibilities have already been described.

One mechanism of carcinogenesis is related with the immune evasion of cancer cells. Cytotoxic T cells have negative regulators such as CTLA-4 and PD-1. The inhibition of these receptors led to the development of multiple immunotherapy bioengineered agents, some of which block PD-1 and CTLA-4 on T cells and others of which block the PD-1 ligand PD-L1 on tumor cells leading the immune system to recognize cancer cells and destroy them. Whole-body PD-1 and PD-L1 PET (imaging the targets of immune checkpoint inhibitor therapy) can be performed using various radionuclides including ^18^F-BMS-98619210 (targeting PD-L1), ^89^Zr-pembrolizumab, ^89^Zr-atezolizumab, ^89^Zr-nivolumab [158,159,160,161], or ^64^Cu-labeled PD-1 and PD-L1 antibodies [162].

The pathophysiology of immunotherapy-related cardiotoxicity is not completely elucidated. Cardiomyocytes express PD1 and PD-L1 [130] and it is supposed that the disruption of the PD1-PDL1 pathway can release autoimmune reactions with immune-mediated cardiac injury and polymorphonuclear leukocyte inflammation [7]. Autoimmune-mediated cardiotoxicity involves myosin-specific T-lymphocytes leading to myocardial cell injury and myocarditis, decreased endothelial nitric oxide synthase and consequently to vascular leak syndrome. On the other hand, mAbs targeting ERBs are related to decreased repair and survival of cardiomyocytes [7]. PET imaging using radiolabeled checkpoint inhibitors might be used to identify patients at risk with high cardiac expression. It would be desirable for clinical trials with these novel PET tracers to also examine cardiac tracer accumulation and possible correlations with immunotherapy-related cardiotoxicity.

CAR-T therapy is a promising form of immunotherapy especially for hematological malignancies [163]. After a sample of a patient’s T cells has been collected from the blood, the cells are re-engineered, so they sprout chimeric antigen receptors on their surface. When these CAR-T cells are reinjected into the patient, the receptors may help the T cells identify and attack cancer cells throughout the body. One major complication of CAR-T cell therapy is the cytokine release syndrome (CRS). CAR-T cells release pro-inflammatory cytokines including interferon gamma (IFNγ), interleukin (IL)-1, IL-2 receptor alpha (RA), tumor necrosis factor alpha (TNFα), and IL-6 to induce a cytotoxic response. The release of these cytokines, particularly IL-6, also plays a role in the pathogenesis of CRS, including the recruitment of additional T-lymphocytes. High levels of circulating IL-6 can also lead to myocardial stunning which may be clinically indistinguishable from septic cardiomyopathy. Furthermore, CRS results in the activation of prostaglandins, which can also impart a risk of cardiotoxic events, such as tachycardia and hypotension. It is also possible that CAR-T therapy results in a direct or “off-target” cardiotoxic injury as a result of cross-reactivity between T cells and Titin [164]. For example, in children receiving CAR-T cell therapy, a rate of approximately 20% suffered from major cardiovascular events [165].

In PET technique, direct in vivo imaging of CAR-T cells is possible. Promising preclinical data suggest that the integration of reporter genes in the CAR-T genome enables their imaging whenever needed [166,167]. For example, if the human natrium iodine symporter is used, the PET tracer tetrafluoroborate can be employed for imaging [2], which is also a promising tracer for thyroid cancer [168]. This enables visualization of the CAR-T cell distribution in the body at a given time, potentially enabling an assessment of organ toxicity and treatment efficacy. Moreover, imaging can be used to visualize the target molecules used in the UniCAR system [169,170]. To overcome the limitations of CAR-T toxicity, the UniCAR system propagates the use of adapter molecules, which link the modified T cell to the cancer cell [169]. The adapter molecule can be conjugated with a radioisotope to visualize the distribution of the UniCAR cells, potentially allowing inferences about side effects [169]. A cardiac-emphasized distribution pattern of CAR-T and UniCAR T cells could be visualized by PET imaging to allow for identification of patients at risk for immunotherapy-related toxicity. Future studies of these PET imaging modalities could also focus on cardiac side effects.

Other exciting tracers that could be used in future for imaging of immunotherapy-related cardiotoxicity are ^18^F-GP1 [171,172], which visualizes activated platelets by targeting glycoprotein IIb/IIIa of aggregated platelets and could therefore be used to show the effects of increased thrombogenic potential, and ^18^F-NaF [173,174], which binds directly to arteriosclerotic plaques. Both have, until now, not yet found their way into clinical routine imaging but might be applicable for imaging of immunotherapy-related cardiotoxicity after systematic clinical investigations.

### 8.2. Technological Perspectives

Over the recent years, a new generation of “digital” PET systems were introduced by all large established PET manufacturers [175,176,177,178,179]. These systems use silicon photomultiplier-based detectors leading to an improved spatial and coincidence timing resolution and reduced image noise [180,181,182,183,184,185]. Possible clinical benefits include a reduction in administered activity or acquisition time [186,187,188,189,190] leading to increased patient comfort, particularly regarding pain-stricken or dyspneic patients [191] from which symptomatic patients with cardiotoxicity might also benefit. In addition, digital PET improves the detectability, especially of small structures and those with low tracer-uptake [182,192,193,194], which may facilitate the visualization of new biomarkers. Moreover, digital PET enhances the applicability of dynamic PET, i.e., evaluation of time-resolved PET data, a technique that can particularly benefit from the improved data quality [195] and will be introduced in the next section.

Next to the application of digital detectors, a recent technical improvement in PET technology was the introduction of total-body PET systems. The extended field-of-view of these systems (≥1 m) allows simultaneous imaging of a significantly larger section of the body than standard PET systems (with a typical field-of-view of 20–30 cm). The increased number of detectors in total-body PET systems further improves sensitivity and image quality compared to “standard” digital systems [196,197,198].

### 8.3. Artificial Intelligence-Based Approaches and Dynamic PET

Advanced image evaluation enables the extraction of clinically relevant information that enables assessment of cardiac function and monitoring of cardiotoxicity. Two aspects of advanced image evaluation will be discussed here.

First, dynamic PET imaging enables the extraction of additional relevant information from a PET scan. Dynamic PET imaging means that the acquisition of PET images begins simultaneously with the administration of the PET tracer. The distinct analysis of time-activity-curves by tracer kinetic modeling in different compartments which are defined within the PET images (compartmental kinetic modeling) allows for a more precise characterization of the uptake process of the investigated tracer [195,199]. From that information, extended conclusions about the investigated disease may be drawn.

For example, if an FDG PET scan is performed as dynamic acquisition, the left ventricular function can be assessed. The first pass of the tracer will be captured in dynamic mode, enabling the quantification of the contractile function. This analysis closely resembles the results of conventional ERNA [200]. Another study could show that dynamic acquisition of ^11^C-Acetate-PET enables the assessment of cardiotoxicity via imaging the myocardial oxidative metabolism. The dynamic acquisitions allow the fit of a kinetic model, which can be used to extract the myocardial oxygen consumption [201]. The possibilities for dynamic imaging will expand even further with the emergence of new technologies such as total-body PET and improved detector technologies that allow for even higher temporal resolution.

Second, novel algorithmic approaches such as methods of machine learning can be used to assess the cardiac function. For example, machine learning could successfully determine the ejection fraction on echocardiograms [202]. In another study, techniques of artificial intelligence were used to predict cancer therapy-related strain alterations [203]. Similar applications might become subjects of future approaches regarding molecular imaging data for investigation of immunotherapy-related cardiotoxicity.

Until now, the application of these approaches to immunotherapy-related changes is still limited. Further studies have to evaluate the value of dynamic PET and machine learning analysis in the context of immunotherapy-related cardiotoxicity.

## 9. Conclusions

Several applications of nuclear cardiology imaging modalities in immunotherapy-related cardiotoxicity patients have found their way into clinical diagnostics. The different examinations reflect technological advances in imaging technique and development of new tracers from blood pool scintigraphy via three-dimensional myocardial perfusion and cardiac sympathetic innervation SPECT to novel elaborate PET technologies, such as FAPI PET for imaging of cardiac remodeling. Table 1 summarizes the most promising nuclear cardiology examination methods that are presented in this review and their most important characteristics. In particular, PET imaging using ^18^F-FDG, ^68^Ga-FAPI, or—depending on the malignancy—^68^Ga-DOTATATE/DOTATOC can allow for simultaneous oncologic staging and cardiac assessment with the benefit of possible early detection of cardiac damage. As the number of immunotherapy patients is increasing, early detection will become even more important to identify patients at risk who demand timely beginning of therapy. For many techniques, the number of clinical applications described so far is small and further investigation is needed before general applicability. On the other hand, due to the ongoing development, the introduction of further exciting tracers, for example, to directly visualize the effect of the therapeutics on the myocardium, can be expected. These might infer further groundbreaking improvements for the application of nuclear cardiology imaging options in immunotherapy-related cardiotoxicity.

## Figures and Tables

**Table 1:** Most promising nuclear cardiology examination methods for assessment of immunotherapy-related cardiotoxicity.

Imaging Modality	Parameters	Advantages	Disadvantages	Publications
FDG PET	Glucose metabolism	− simultaneous oncologic staging/cardiac assessment − early detection of cardiac damage − widely available	− can be non-specific − laborious patient preparation	Case reports
FAPI PET	Cardiac remodeling	− simultaneous oncologic staging/cardiac assessment − early detection of cardiac damage − highly specific − simple patient preparation	− limited to large centers	Case reports
DOTATOC/ DOTATATE PET	SSTR *-Expression	− early detection of cardiac damage − simple patient preparation − widely available	− simultaneous oncologic staging/cardiac assessment limited to SSTR *-expressing malignancies	Case reports
MIBG SPECT	Sympathetic innervation	− potential early detection of cardiac damage − simple patient preparation − widely available	− not yet described for immunotherapy patients	-
Myocardial perfusion SPECT	LVEF ^†^ wall motion perfusion (stress/rest)	− highly standardized − widely available − simultaneous assessment of CAD ^‡^ and HF ^§^ − value in CAD ^‡^ evidenced by a very high number of studies − cost-effective	− no early detection	Case reports
ERNA	LVEF ^†^ wall motion	− highly standardized − widely available − excellent reproducibility − cost-effective	− no early detection	-

* SSTR: somatostatin receptor. ^†^ LVEF: left ventricular ejection fraction. ^‡^ CAD: coronary artery disease. ^§^ HF: heart failure.
